# Candidate Anti-COVID-19 Medicinal Plants from Ethiopia: A Review of Plants Traditionally Used to Treat Viral Diseases

**DOI:** 10.1155/2021/6622410

**Published:** 2021-06-04

**Authors:** Dires Tegen, Kindalem Dessie, Destaw Damtie

**Affiliations:** ^1^South Gondar Zone, Dera Woreda Education Office, Dera, Ethiopia; ^2^Bahir Dar University, College of Sciences, Department of Biology, Bahir Dar, Ethiopia

## Abstract

**Background:**

Emerging viral infections are among the major global public health concerns. The pandemic COVID-19 is a contagious respiratory and vascular disease caused by severe acute respiratory syndrome coronavirus 2 (SARS-CoV-2). There are no medicines that can treat SARS-CoV-2 except the vaccines. Therefore, searching for plant-originated therapeutics for the treatment of COVID-19 is required. Consequently, reviewing medicinal plants used to treat different viral infections is mandatory. This review article aims to review the ethnobotanical knowledge of medicinal plants traditionally used to treat different viral diseases by the Ethiopian people and suggests those plants as candidates to fight COVID-19.

**Methods:**

Articles written in English were searched from online public databases using searching terms like “Traditional Medicine,” “Ethnobotanical study,” “Active components,” “Antiviral activities,” and “Ethiopia.” Ethnobotanical data were analyzed using the Excel statistical software program.

**Result:**

From the 46 articles reviewed, a total of 111 plant species were claimed to treat viral infections. Fifty-six (50.4%) of the plant species had reported to have antiviral active components that are promising to treat COVID-19. Lycorine, gingerol shogaol, resveratrol, rhoifolin, oleanolic acid, kaempferol, rosmarinic acid, almond oil, ursolic acid, hederagenin, nigellidine, *α*-hederin, apigenin, nobiletin, tangeretin, chalcone, hesperidin, epigallocatechin gallate, allicin, diallyl trisulfide, ajoene, aloenin, artemisinin, glucobrassicin, curcumin, piperine, flavonoids, anthraquinone, hydroxychloroquine, and jensenone were some of them.

**Conclusion:**

The Ethiopian traditional knowledge applies a lot of medicinal plants to treat different viral infections. Reports of the chemical components of many of them confirm that they can be promising to fight COVID-19.

## 1. Introduction

Viral diseases are responsible for the global morbidity and mortality of human beings [1]. The pandemic COVID-19 is among such viral outbreaks challenging the healthcare systems around the world [2]. From 31 December 2019 to 31 October 2020, this pandemic resulted in 45,667,780 cases and 1,189,499 deaths globally and 95,789 cases and 1,464 deaths in Ethiopia [3]. However, no specific medications and drugs are known to treat this viral disease. Consequently, reports show that people from different countries use medicinal plants for the prevention and treatment of COVID-19, although not confirmed by the World Health Organization (WHO) for safety issues [4]. Because they contain various active components, medicinal plants can be alternatives to prevent and combat COVID-19 [5].

Plant secondary metabolites like lycorine [6], gingerol shogaol [7], resveratrol rhoifolin [8], oleanolic acid [9], kaempferol [10], rosmarinic acid [11], almond oil [12], ursolic acid [11], hederagenin, nigellidine, and *α*-hederin [11, 13], apigenin, ethyl cholate, nobiletin, tangeretin, chalcone, and hesperidin [10, 14, 15], epigallocatechin gallate [16], allicin, diallyl trisulfide ajoene, and apigenin [14, 17], aloenin [18], artemisinin [6, 19], glucobrassicin [10, 11], apigenin [11], curcumin [20], piperine [12], flavonoids, anthraquinone, and hydroxychloroquine [21], and jensenone [22] are reported to have antiviral activities. The mechanism of action of these secondary metabolites may be due to their greater binding affinity for SARS-CoV-2 6LU7 and 6Y2E proteases and inhibition of SARS-CoV-2 M protease (Mpro) and Spike (S) glycoprotein [6–22].

Globally, millions of people rely on medicinal plants not only for their primary healthcare systems but also for income generation and livelihood improvement [23]. Moreover, at least 25% and 50% of the pharmacopeia are derived from plant products and are originated from natural products, respectively [24]. Nowadays, traditional healers from different habitats and geographical locations are showing new candidate combinations for the treatment of viral infections such as SARS-CoV [5].

Using traditional medicine has a long history in Ethiopia. About 80% of the Ethiopian population is still dependent on the use of folk medicine [25–27], due to its cultural acceptability, economic affordability, and efficacy against certain types of diseases compared to modern medicine [28]. However, the plants and the associated indigenous knowledge in the country are gradually declining because of environmental degradation, deforestation, lack of documentation, and potential acculturation [29].

Common cold, influenza, and COVID-19 share common characteristics. All of them affect the respiratory tract and have modes of transmission: direct contact, droplets, and fomites. Cough, sneezes, fever, shortness of breath, sore throat, and headache are among the common symptoms of these diseases [30]. Traditional healers from Ethiopia use medicines of plant origin to treat viral infections like the common cold, rabies, influenza, herpes simplex, herpes zoster, and hepatitis. Due to their fewer side effects, better patient tolerance, and relatively low cost, the use of medicinal plants is a common practice by the Ethiopian people.

Due to its ecological and cultural diversity, Ethiopia is a rich source of herbal medicine [31]. Plant extracts contain a lot of active components, so they have a wide range of activities against microorganisms. That is, they act on multiple active sites of the pathogen [32]. Therefore, a medicinal plant used to treat one viral infection may serve to fight other viral infections. This review, therefore, focuses on the identification of medicinal plants used by traditional healers of Ethiopia to treat viral diseases and extrapolates this knowledge for the fight of COVID-19.

## 2. Methods

### 2.1. Study Design and Setting

The location of Ethiopia is in the horn of Africa. Its boundaries are Eritrea to the North, Djibouti and Somalia to the East, Sudan and South Sudan to the West, and Kenya to the South. The current UN report shows that the Ethiopian population is estimated to be 115,855,859. Ethiopia's population is equivalent to 1.47% of the world's population. Around 21.3% of the population is an urban community. The population density in Ethiopia is 115/km^2^ (298 people/mi^2^) [33].The total land area is 1,104,300 km^2^ [34].

### 2.2. Search Strategies

The authors explored articles from PubMed, ScienceDirect, and Web of Science search engines using the following core search terms and phrases: “Traditional Medicine,” “Ethnobotanical study,” “Active components,” “Antiviral activities,” and “Ethiopia.” We used the search terms separately and in combination with Boolean operators like “OR” or “AND.” Besides, we searched for gray literature through the review of available references. Searching for relevant literature included in this systematic review was conducted from September 2020 to October 2020.

### 2.3. Inclusion and Exclusion Criteria

Studies that were written in the English language, reporting about the antiviral activity of traditional medicines, phytochemical analysis of medicinal plants, and candidate anti-COVID-19 medicinal plants in Ethiopia, Africa, China, Europe, and Western countries, were retrieved and included in this study. However, we excluded studies that did not contain antiviral medicinal plants.

### 2.4. Data Extraction

All authors contributed to the data extraction protocol preparation and evaluation. The data extraction protocol consists of the scientific, family, and local names, parts used, preparation methods, administration routes, diseases treated, and references.

### 2.5. Data Analysis

Ethnobotanical data were entered in an Excel spreadsheet and analyzed using Excel statistical software program. We tabulated and compiled quantitative data using descriptive statistics to identify the number and percentage of species and families of antiviral plants and expressed them in tables.

## 3. Results and Discussion

### 3.1. Search Results

**Figure 1:**
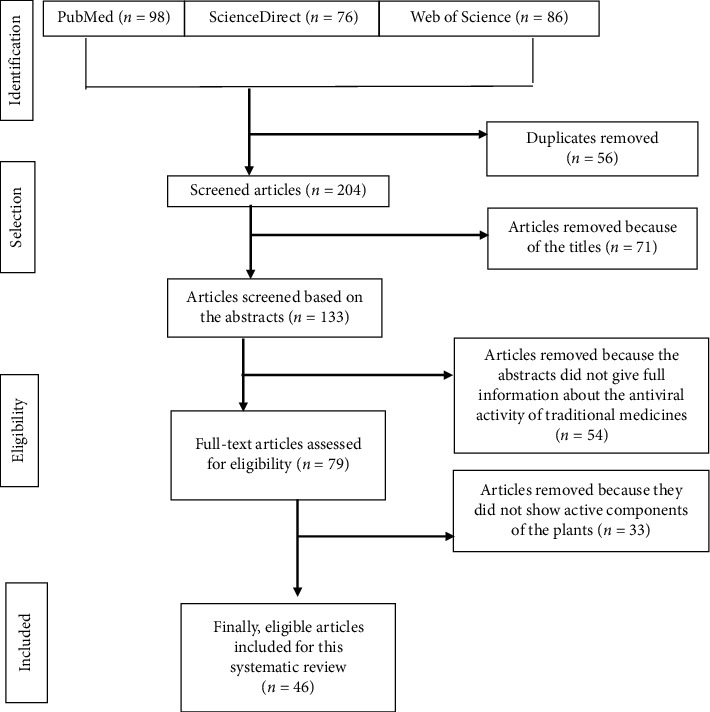
PRISMA flowchart of the reviewed articles on antiviral medicinal plants from Ethiopia.

From the total of 260 articles retrieved, only 46 (17.7%) of the studies met the eligibility criteria (Figure 1).

### 3.2. Identified Plants with Antiviral Activities

From the 46 articles reviewed, 111 plant species claimed to treat eleven viral infections. The most frequently reported viral diseases to be treated by the 111 plants were rabies (reported 36 times), hepatitis (30 times), common cold (26 times), herpes zoster (17 times), influenza (10 times), Herpes simplex virus (8 times), Wart (6 times), HIV-1 (5 times), Bursal viral diseases (once), flu (once), and Smallpox (once) (Table 1).

### 3.3. Taxonomic Diversity of Medicinal Plants Used for the Treatment of Viral Diseases in Ethiopia

We reviewed 162 plants which were grouped under 111 species and 57 families (Table 2). Among the families, Fabaceae was represented by 8 (7.2%) species, Solanaceae and Lamiaceae by 6 (5.4%) species each, Euphorbiaceae and Asteraceae by 5 (4.5%) species each, and Meliaceae, Vitaceae, Apiaceae, Anacardiaceae, Moraceae, Oleaceae, Cucurbitaceae, Rutaceae, and Acanthaceae by 3 (2.73%) species each, and the remaining 43 families were represented by 1 to 2 species (Table 2).

Solanaceae was represented by *n* = 12, 7.41% plants, followed by Euphorbiaceae (by *n* = 11, 6.8% plants), Fabaceae and Lamiaceae (by *n* = 9, 5.6% plants each), Alliaceae and Phytolaccaceae (by *n* = 8, 4.9% plants each), Acanthaceae (by *n* = 7, 4.3% plants), Myrtaceae and Zingiberaceae (by *n* = 6, 3.7% plants each), Asteraceae and Moraceae (by *n* = 5, 3.09% plants each), and the remaining 43 families by 1 to 4 plants (Table 2).

### 3.4. Medicinal Plants with Antiviral Active Components

A range of active compounds with potential antiviral agents for future drug development has been identified from plants [77]. People in Ethiopia use different medicinal plants to treat different viral infections even without knowing their active components (Table 1). However, different literature shows that 56 (50.4%) of the plants reviewed contained components with antiviral activity (Table 3).

Flavonoids are secondary metabolites with antiviral properties [99]. The Ethiopian medicinal plants *Acacia abyssinica, Acacia etbaica,* and *Acacia nigra* [5], *Moringa borziana* [21]*, Acanthus polystachyus* [78], *Azadirachta indica* [81], and *Osyris quadripartite* [91] were reported to contain flavonoids.

Reports show that tannins block virus attachment, entry, and cell-to-cell spread by binding to viral glycoproteins on viruses and the surfaces of infected cells [100]. The Ethiopian medicinal plants *Acacia abyssinica, Acacia etbaica,* and *Acacia nigra* [5] and *Acanthus polystachyus* [78] are reported to have tannins so that they can be good candidates to fight COVID-19.

Many terpenoids of plant origin have antiviral activities against severe acute respiratory syndrome coronavirus [101]. Medicinal plants reviewed in the present study may possess terpenoids. Studies among some of these medicinal plants show that they possess these secondary metabolites. Some of the medicinal plants with terpenoid active components were *Acacia abyssinica, Acacia etbaica,* and *Acacia nigra* [5] and *Osyris quadripartite* [91].

Polyphenols have demonstrated potent antiviral activities. For example, the polyphenol in green tea controls viruses such as hepatitis C, chikungunya, hepatitis B, herpes simplex virus type 1, influenza A, vaccinia, adenovirus, reovirus, vesicular stomatitis, and Zika (ZIKV) [102]. *Acacia abyssinica, Acacia etbaica,* and *Acacia nigra* [5], *Acanthus polystachyus* [78], and *Azadirachta indica* [81] of the present review contained polyphenols in their extracts.

*Acanthus polystachyus* [78] contained saponins that possess various biological activities, including antiviral action [103]. *Ocimum basilicum*, *Ocimum lamiifolium*, *Ocimum urticifolium*, and *Olea europaea* subsp. *cuspidate* [11], *Osyris quadripartite* [91], and *Acokanthera schimperi* [79] contain ursolic acid which is a pentacyclic triterpenoid with potent antiviral activities [104].

Another plant secondary metabolite with antiviral activity is oleanolic acid [105]. It is reported from *Syzygium aromaticum* [9], *Ocimum basilicum*, *Ocimum lamiifolium*, *Ocimum urticifolium*, and *Olea europea subsp cuspidate* [11], *Osyris quadripartite* [91], *Acokanthera schimperi* [78], *Dregea schimperi* [88], *Euphorbia abyssinica* [89], and *Phytolacca dodecandra* [93]. Oleanolic acid has a binding affinity for SARS-CoV-2 M protease and Spike (S) glycoprotein [106].

The plant metabolite quercetin inhibits viral entry into target cells via interaction with viral HA protein [107]. Medicinal plants from Ethiopia, *Allium cepa* [16], *Lepidium sativum* [22], *Azadirachta indica* [81], *Osyris quadripartite* [91], *Amaranthus hybridus Linn* [80], *Clematis hirsute* [84], *Carissa edulis* [90], *Ricinus communis* [95], and *Ruta chalepensis* [13], are reported to contain quercetin.

Epigallocatechin-3-O-gallate (EGCG) is known to inhibit a variety of DNA and RNA viruses [108]. It is found in *Camellia sinensis* [10] and *Allium cepa* [16]. Allicin exhibits antiviral, antifungal, and antiparasitic activities [109]. This phytochemical is reported from *Allium sativum* [14, 17], a medicinal plant used to treat viral infections by people in Ethiopia.

In vitro and in vivo results show that apigenin exhibits antiviral activities [110]. It is found in *Capsicum annuum* [11], *Citrus aurantium* [5, 10, 14, 15], *Citrus limon* [5, 10, 14, 15], and *Allium cepa* [14, 17]. Reports show that kaempferol has antiviral activities against influenza A virus (H1N1 and H9N2), human immunodeficiency virus (HIV) 1, and JEV [111]. Many medicinal plants used to treat viral infections in Ethiopia such as *Citrus aurantium* L.*, Citrus limon* (L.) Burm. f*., Capsicum annuum* L.*, Eucalyptus globulus, Osyris quadripartite, Amaranthus hybridus* Linn.*, Clematis hirsute, Ricinus communis* L.*, Ruta chalepensis* L.*, Carissa edulis, Phaseolus vulgaris* also contain this active component [10, 11, 13, 22, 80, 83, 84, 91, 92, 95].

Lycorine is a compound with broad antiviral activity. It is reported to possess anti-SARS-CoV activity [6]. It is possessed in Ethiopian medicinal plants traditionally used to treat viral infections, for example, in *Crinum abyscinicum* Hochst. ex A. Rich. [57].

## 4. Conclusions

Traditional healers in Ethiopia have knowledge of medicinal plants with potential antiviral activity. Literature shows that the majority of the plants prescribed by traditional healers in Ethiopia have antiviral compounds. Therefore, these medicinal plants should be researched for anti-COVID-19 properties.

## Figures and Tables

**Table 1:** The medicinal plants used to treat viral diseases in different parts of Ethiopia.

No.	Scientific name	Family	Local name	Parts used and preparation method	RA	DT	Ref.
1	*Acacia abyssinica* Hochst. ex Benth.	Fabaceae	Memona (Tig)	Crush the bark and apply on the affected part	Dermal	H. zoster	[35]
2	*Acacia etbaica* Schweinf.	Fabaceae	Seraw (Amh)	Crushed bark	Oral	Wart	[36]
3	*Acacia nigra*	Fabaceae	Tikur grar (Amh)	Crush or pound and squeeze the leaf and apply on allergic skin	Dermal	H. zoster	[37]
4	*Acanthus polystachyus*	Acanthaceae	Kucheshile (Amh)	Crush the root and pound and give with water	Oral	Rabies	[36, 38]
5	*Acokanthera schimperi* (A. DC.) Schweinf.	Apocynaceae	Meriz (Amh)	Roots are burned on fire and fumigated	Dermal	Hepatitis	[38, 39]
6	*Allium sativum*	Alliaceae	Nechsenkret (Amh)	(i) Crushed rhizomes are pounded and eaten with honey (ii) Crush the bulb and drink with water	Oral	Influenza virus	[36, 38]
				(i) Bulb is pounded and mixed with meat soup and used as a drink (ii) Boiled bulb vapor is inhaled orally and nasally (iii) Cloves ground up and mixed with honey, take first thing in the morning on an empty stomach	(i) Oral (ii) Oral and nasal (iii) Oral	C. cold	[40–43]
7	*Allium cepa*	Alliaceae	QeY shikurt (Amh)	Eat the part of the bulb with other foods	Oral	Rabies	[35]
				Crush the bulb and drink with water	Oral	HSV	[44]
8	*Aloe macrocarpa* Tod.	Aloaceae	Eret (Amh)	Leaf of *A. macrocarpa* is powdered and mixed with honey	Oral	Wart	[45]
9	*Amaranthus hybridus* Linn.	Amaranthaceae	Tenbelel (Amh)	Crush the fruit and leaf of *Amaranthus hybridus*	Oral	Hepatitis	[36]
10	*Argemone mexicana* L.	Papaveraceae	Yahyaeshoh (Amh)	(i) Crush the leaf (ii) Crush the root and give with water	Oral	Influenza, Rabies	[36, 38]
11	*Artemisia afra* Jack. ex Willd. and *Artemisia annua* L.	Asteraceae	Chikugn (Amh)	Grind leaves and apply topically	Dermal	Smallpox	[40]
				Crushed and powdered leaf	Nasal, oral	Influenza	[36]
12	*Azadirachta indica.* A. Juss.	Meliaceae	Neem (Amh)	Leaves	Oral	HIV-1	[46]
13	*Bersama abyssinica* Boyle	Melianthaceae	Azamer (Amh)	Bark/leaves/roots		Rabies, HIV-1	[47]
14	*Brassica carinata* A. Br. Herb	Brassicaceae	Gomen (Amh)	The dried leaf was powdered and mixed with water then drunk	Oral	C. cold	[48]
15	*Brucea antidysenterica* J. F. Mill	Simaroubaceae	Waginos (Aballo)(Amh)	Fresh parts of the stem are boiled in water and the steam is inhaled through the mouth and nose	Oral & nasal	Hepatitis	[49]
				Squeeze the whole part of the plant and bake with teff flour and give for 3 days (together with *Croton macrostachyus* and *Rumexnervosus*)	Oral	Rabies	[50]
16	*Calpurnia aurea* (Ait.) Benth.	Fabaceae	Digita (Amh)	Crush the seeds of *Calpurnia aurea* and mix with water	Orally	Rabies	[51]
17	*Camellia sinensis*	Theaceae	Shay kitel (Amh)	Drink the leaves with tea	Oral	HBV, HCV, influenza, HIV, BCV	[52, 53]
18	*Capsicum annuum* L.	Solanaceae	Berbere (Amh)	Pounded being mixed with the leaf of *V. sinaiticum, O. quadripartita, C. aurea* (concoction), then bandage on the wound	Dermal	H. zoster	[37]
19	*Carica papaya* L.	Caricaceae	Papaya (Amh)	Fresh fruit and leaf pounded and crushed, add water	Oral	H. zoster,	[37, 44]
20	*Carissa edulis* Vahl.	Apocynaceae	Agam (Amh)	The root is powdered and mixed with food	Oral	Rabies	[54]
21	*Cayratia ibuensis* (Hook.f.) Suess.	Vitaceae	Udusalim Rumiyi (Oro)	The roots crushed and pounded, then boiled and drink 2-3 cups of coffee in the morning	5–7 of tea spoons drink (oral)	Hepatitis	[55]
22	*Centella asiatica* L.	Apiaceae	Yeait joro (Amh)	A quarter of a finger-sized root is ground, mixed with water, filtered, and taken orally	Oral	Rabies	[39]
23	*Citrus aurantium* L.	Rutaceae	Bahir Lome (Amh)	Squeezing fruit (juice)	Oral	C. cold	[43]
24	*Citrus limon* (L.) Burm. f.	Rutaceae	Lome (Amh)	Squeezed fruit (juice)	Oral	C. cold	[36]
25	*Clematis hirsute* Perr. & Guill.	Ranunculaceae	Hareg (Tig)	Burn leaves in oven with leaves of *Dodonaea angustifolia*, grind, mix with butter and apply on the affected part.	Dermal	H. zoster	[35]
26	*Clutia abyssinica* Jaub. & Spach.	Euphorbiaceae	Tewshealalito (Tig) Fiyle feji (Amh)	Dry and mix leaves with dried leaves of *Calpurnia aurea* and *Datura stramonium*, grind, add butter, and spread the paste on the affected part (i) Crush the root and boiled with water (Decoction)	Dermal Oral	H. zoster, hepatitis	[35, 37]
27	*Coffea arabica* L.	Rubiaceae	Bunna (Amh)	Boil the leaf, decant then drink the juice	Oral	C. cold	[38]
28	*Combretum collinum*	Combretaceae	Abalo (Amh)	The seed of *Combretum collinum* with the seed of *Solanumda syphyllum* are crushed together powdered, mixed with “tella” and drunk for 3 days	Oral	Rabies	[50]
29	*Coriandrum sativum*	Apiaceae	Dimblal (Amh)	—	—	Potential anti-COVID-19	[56]
30	*Cordia africana*	Boraginaceae	Wanza (Amh)	Boiled with sorghum (decoction) and drinking	Oral	Hepatitis	[37]
31	*Crinum abyscinicum* Hochst. ex A. Rich.	Amaryllidaceae	Yejib shinkurt (Amh)	Bulb of *Crinum abyscinicum* is used to treat rabies	Oral	Rabies	[57]
32	*Crotalaria incana* L.	Fabaceae	Atarii Kuruphee (Oro)	Sap from the whole part of the plant is directly creamed on affected area	Dermal	Hepatitis	[41]
33	*Croton macrostachyus* Del.	Euphorbiaceae	Bisana (Amh)	(i) Shoots are crushed with water, filtered and the solution is taken orally (for hepatitis) (ii) The fresh root bark is crushed, pounded, mixed with water, and given orally (for rabies)	Oral	Hepatitis, rabies	[39, 44, 51]
34	*Cucumis ficifolius* A. Rich.	Cucurbitaceae	Yemidir Embuay (Amh)	Crushed fresh root with water fermented for 3 days is taken with honey early morning before breakfast orally until the cure	Oral	Rabies	[49]
35	*Cucurbita pepo* L.	Cucurbitaceae	Hamham (Tig)	Fresh leaf boiled with water and given orally	Oral	Influenza	[44]
36	*Curcuma longa*	Zingiberaceae	Erd (Amh)	—	—	HBV, HCV	[58]
37	*Cussonia ostinii* Chiov.	Araliaceae	Harfattu (Oro)	Bark (root) of *Cussonia ostinii*, leaf *Asplenium monathes* and the leaf of *Calpurnia subdecandra* were pounded together, and 2 cups were given to cattle	Orally	Hepatitis	[41]
38	*Cyphostemma adenocaula* (A. Rich.)	Vitaceae	Asserkush (Amh)	The root was boiled with milk and filtered and the filtrate was taken in an empty stomach full of a coffee cup daily for 3 consecutive days	Orally	Rabies	[50]
39	*Datura stramonium* L.	Solanaceae	Atsefaris (Amh)	Leafy stem is squeezed and its drop prepared with butter	Dermal	Wart	[35]
				Leafy stem is squeezed and its drop prepared with butter	Dermal cream	Wart	[36]
				Crushed and homogenized leaves drunk with water	Oral	Rabies	[50, 59]
				Dried leaves of the plant and *Calpurnia aurea* and *Clutia abyssinica* are ground, mix powder with butter, and apply on the affected part	Dermal	H. zoster	[35]
40	*Diplolophium africanum* Turcz.	Apiaceae	Zegerawta (Amh)	Pound the root and give with water	Orally	Rabies	[38]
41	*Dipsacus pinnatifidus* Steud. ex A. Rich.	Dipsacaceae	Fereze ng/kelem (Amh)	Pound the leaf and give with water	Nasal	Rabies	[38]
42	*Dodonaea angustifolia* L.f.	Sapindaceae	Kitkita (Amh)	Dry the leaf of the plant alone or mix with the leaf of *Clematis hirsuta* on a hot stove, grind, add butter and rub the affected part	Dermal	H. zoster	[35]
43	*Dorstenia barnimiana* Schweinf.	Moraceae	Work Bemeda (Amh)	Root powder with shimmed milk or nug is taken orally early morning until a cure	Orally	Rabies	[49]
				Root powder with shimmed milk or nug is taken orally early morning until a cure	Orally	Hepatitis	[49]
44	*Dregea rubicunda* Schum.	Asclepiadaceae	Kuandira (Amh)	Crush and drink with milk	Orally	Rabies	[38]
45	*Dregea schimperi* (Decne.) Bullock.	Asclepiadaceae	Shanqoq (Tig)	Crush and drink the fluid	Orally	Rabies	[35]
46	*Echinops amplexicaulis* Oliv.	Asteraceae	Kosorru Hare (Oro)	The root of *Echinops amplexicaulis is* dried, powdered, and mixed with water The concoction is given to cattle	Orally	Hepatitis	[41]
47	*Ekebergia capensis*	Meliaceae	…	The leaf of *Ekebergia capensis* is crushed and add water	Orally	C. cold	[36]
48	*Eucalyptus globulus* Labill.	Myrtaceae	Nech bahirzaf (Amh)	Boil and fumigate with the fume	Nasal, oral, and dermal	C. cold	[36]
				(i) Leaf of *Eucalyptus globulus* is chopped and boiled; the steam bath is taken by humans; vapor inhaled orally and nasally (ii) Boil *Eucalyptus* and Damakasse in water and inhale (iii) Leaf of *E. globulus* is boiled in water	Nasal, orally	Influenza	[35, 40–42, 45]
49	*Euphorbia abyssinica* G.F.Gmel.	Euphorbiaceae	Kulkual (Amh)	Stems are burned on fire and fumigated	Dermal	Hepatitis	[39]
				Mix the latex *of Euphorbia abyssinica* with milk and drink it	Orally	Rabies	[38]
50	*Ficus sycomorus* L.	Moraceae	Sholla (Amh)	(i) The sap of *Ficus sycomorus* is creamed directly on the skin (for hepatitis) (ii)The bark of *Ficus sycomorus* and root of *Prunus africana* are powdered together and backed with teff flour and eaten (for rabies)	Dermal Oral	Hepatitis, rabies	[41, 45]
51	*Ficus* sp.	Moraceae	Warka (Amh)	The stem bark and the latex are mixed with *Phytolacca dodecandra* (leaf) and given	Oral	Rabies	[51]
52	*Gnidia stenophylla* Gilg.	Trymalaceae	Katarichaa (Oro)	The decoction of the root is taken with goat milk	1 teaspoon drink orally	Hepatitis	[55]
53	*Hypoestes forskaolii* (Vahl) R.Br.	Acanthaceae	Girbia (Tig)	A bunch of leaves was collected from 7 different sites, mixed with 10 tin cans of water, stored for 7 days, and washed for 7 consecutive days	Dermal	H. zoster	[60]
54	*Jasminum abyssinicum* Hochst.	Oleaceae	Tembelel (Amh)	Pounded being mixed with the leaf of *V. sinaiticum*, *O. quadripartita, C. aurea, S. uliginosa, D. stramonium,* and *P. schmperi*	Dermal	H. zoster	[37]
55	*Jatropha curcas* L.	Euphorbiaceae	Yesudan-gulo (Amh)	Crush the seed of *Jatropha curcas* mixed with water	Orally	Rabies	[51]
56	*Justicia schimperiana* (Hochst. ex Nees) T. Anders	Acanthaceae	Smiza (Amh)	(i) Root and leaf of *Justica schimperiana* are pounded together and mixed with water and 2-3 cups of tella are used as a drink (ii) Seed of *J. Schimperiana* is crushed and mixed with water and filtered (iii) The *Justicia schimperiana* and *Brucea antidysenterica* leaves are used to treat rabies	Oral	Rabies	[36, 41, 45, 59, 61]
				Sniff unprocessed or after rubbing	Nasal	C. cold	[36]
				(i) Juice of seven shoot meristems that can be mixed with fresh water and drink a cup of the mixture (ii) Juvenile leaf of *Justicia schimperiana* boiled with milk (decoction)	Orally	Hepatitis	[37, 62]
57	*Laggera integrifolia* Sch. Bip. ex A. Rich	Asteraceae	Gimmie (Amh)	The leaf is inhaled sometimes through the nose	Nasal (nostril)	C. cold	[63]
58	*Lens culinaris* Medic.	Fabaceae	Misir (Amh)	Dry seeds are ground, powder is soaked in water, and cream is smeared on the affected part	Dermal	H. zoster	[39]
59	*Lippia abyssinica*	Lamiaceae	Koseret (Amh)	—	Nasal	C. cold	[59]
60	*Lobelia rhynchopetalum* Hemsl.	Lobeliaceae	Jibara (Amh)	Roots are ground, mixed with milk, and solution drunk for five days	Orally	Rabies	[39]
61	*Lycopersicon esculentum* (L.) Mill.	Solanaceae	Timaatima (Oro)	Fresh fruit put in the fire and eaten when getting hot in order to get relief from the common cold	Oral	C. cold	[48]
62	*Mangifera indica*	Anacardiaceae	Mango (Amh)	Bark/leaves	Oral	C. cold, HSV-1/2	[46]
63	*Millettia ferruginea* (Hochst.) Bak.	Fabaceae	Birbira (Amh)	Heat stick, then touch their body with hot part	Dermal	Rabies	[38]
64	*Moringa borziana* Mattei Mawe	Moringaceae	Tamergnaw ketel (Shiferaw) (Amh)	Leaf chewing	Chewing Oral	C. cold	[36]
65	*Musa* spp.	Musaceae	Muz (Amh)	—	—	SARS-CoV-2, influenza	[64, 65]
66	*Myrica salicifolia* Hochst. ex A. Rich.	Myricaceae	Shinet (Amh)	Crush, powder, then sniff	Nasal	C. cold	[38]
67	*Nicandra physalodes* (L.) Gaertn	Solanaceae	Hawwixii (Oro)	*Nicandra physalodes (L.)* Gaertn roots are pounded and mixed with cold water; 2–4 cups of tella are used as a drink	Oral	Hepatitis	[41]
68	*Nicotiana tabacum*	Solanaceae	Tamiba (Had)	Dry leaves are pounded and powdered, then drunk or smelled through the nose of humans	Nasal	C. cold	[43]
69	*Nigella sativa*	Ranunculaceae	Tikur Azmud (Amh)	Fried seeds wrapped in a piece of cloth and sniffed three times daily, wrap in small leaf, stick up nose	Orally Nasal	C. cold	[40, 62]
70	*Ocimum basilicum* L. Herb	Lamiaceae	Bessobla (Amh)	Fresh leaves together with the root of *Aloe macrocarpa* concocted together and drink the solution	Oral	Flu, CVB1	[48]
71	*Ocimum lamiifolium* Hochst. ex Benth.	Lamiaceae	Damakassie (Amh)	Crushed and mixed/concocted/with coffee and take	Orally	C. cold	[59]
				(i) Squeeze leaves and drink the juice with coffee, or apply the rubbed leaves into the nose	Nasal	Influenza and acute viral infection	[42, 66]
72	*Ocimum urticifolium* Roth.	Lamiaceae	Dama kesie (Amh)	Boil with tea and drink	Orally	C. cold	[38]
73	*Olea europaea* subsp. *cuspidate*	Oleaceae	Weyra (Amh)	Boiled, adding salt for the night and isolate the residue (decoction)	Orally	Hepatitis	[37]
74	*Olinia rochetiana* A. Juss	Oliniaceae	Noole (Sid)	The leaf is heated slightly, rubbed by the hands, and then inhaled through nostrils	Nasal	Viral common cold	[66]
75	*Osyris quadripartita* Decn.	Santalaceae	Keret (Amh)	Dried and pounded then 2 spoonsful powder is mixed with a cup of water, drink for 3 consecutive days	Orally	Hepatitis	[37]
				Pounded being mixed with the leaf of *C. annuum, V. sinaiticum, C.aurea, J. abyssinicum* (concoction)	Dermal	H. zoster	[37]
76	*Otostegia integrifolia* Benth.	Lamiaceae	Tunjut (Amh)	Smoking and fumigating the house	Smoking, oral	C. cold	[36, 38]
77	*Piper nigrum*	Piperaceae	Kundo berbere (Amh)	—	—	VSV, PIV, CVB3	[67]
78	*Phaseolus vulgaris*	Fabaceae	Bakela (Amh)	—	—	HIV-1, RSV, and HSV-1	[68, 69]
79	*Phytolacca dodecandra*	Phytolaccaceae	Endod (Amh)	(i) Root is crushed and pounded, mixed with water; one-third of the tella cup is given to humans (liver problem); *Phytolacca dodecandra* root is crushed and pounded, mixed with water; one-third of a cup is given to humans (ii) Dried root of *Phytolacca dodecandra* powder and one-two cups of domestic alcohol (malakia) are taken orally (for rabies) (iii) Chopped root and leaves mixed with honey are given orally (for rabis) (iv) Fresh root of *Phytolacca dodecandra* is pounded, mixed with water, one arake glass of the solution is given for 7–10 days (for humans)	Oral	(i) Liver problem (hepatitis), (ii) Rabies	[41, 42, 48, 70]
				(v) Squeeze and apply on the wounded part	Dermal	H. zoster	[37]
				Juice extracted by pounded fresh root mixed with milk of similar cow and calf Roots are chewed and fluid swallowed; as an antidote, *Guizotia abyssinica* solution is taken orally	Orally	Rabies	[39]
				Juice of crushed fresh root taken with skimmed milk	Oral	Rabies	[44]
				Juice of crushed fresh root taken with skimmed milk	Orally	Hepatitis “wef beshita'	[49]
80	*Plantago lanceolata* L.	Plantaginaceae	Korxobi (Oro)	(i) The leaf is squeezed and apply on the affected dermal part (ii) The squeezed leaf is pasted with butter and made to ointment	Dermal	Wart, herpes wounds	[54]
81	*Podocarpus falcatus*	Podocarpaceae	Birbirsa (Oro)	Fresh stem barks boiled and filtered and then drunk in the middle of the night for three days; dry stem bark crushed and pounded then parted on the wound	Oral	Jaundice (hepatitis) or rabies	[43]
82	*Podocarpus gracilior*	Podocarpaceae	Zigba (Amh)	Combined Zigba (*Podocarpus gracilior)* of Dokuma (*Syzgium guineense*, listed next) in a cold maceration; drink on an empty stomach first thing in the morning, this induces vomiting which is thought to help treat Yellelitwofe (hepatitis)	Oral	Yellelito wofe (hepatitis)	[40]
83	*Polygala obtusissima* Chod.	Polygalaceae	Calmala (Afa)	The fresh leaves are pounded, kept in a handkerchief, and inhaled	Inhalation (nasal)	C. cold	[71]
84	*Prunus dulcis*	Rosaceae	Lewuz (Amh)	Drink with tea	Oral	HSV-1/ 2	[72]
85	*Rhus natalensis*	Anacardiaceae	Debobosha (Amh)	Pounded being mixed with *J. abyssinicum, D. stramonium,* and *S. nigrum* (concoction); wash the entire body first and apply the remedy on the wound	Dermal	H. zoster	[37]
86	*Ricinus communis* L.	Euphorbiacea	Kabosimbiro (Oro)	Fresh leaves are crushed and mixed with water and one cup of tea is taken for 3 consecutive days	Orally	Rabies	[50]
				(i) The root is pounded, well-spiced, and mixed with food (ii) Freshly pounded and squeezed leaves of *Ricinus communis* L. with milk for treating patients of rabies	Oral	Rabies	[54, 73]
87	*Rosa abyssinica*	Rosaceae	Qega (Amh)	—	Oral	Enteric coronavirus.	[74]
88	*Rosmarinus officinalis*	Lamiaceae	Tibs kitel (Amh)	—	—	RSV-A and B	[75]
89	*Rumex abyssinicus*	Polygonaceae	Mekmoko (Amh)	Root decocted, drunk or chewed	Oral	Hepatitis	[40]
90	*Rumex crispus*	Polygonaceae	Enbacho (Amh)	Roots chewed and juice swallowed	Oral	Hepatitis	[40]
91	*Ruta chalepensis* L.	Rutaceae	Tena adam (Amh)	Leaf of *Ruta chalepensisis* pounded with the bulb of *Allium sativum* mixed with soup and used as a drink	Oral	Influenza	[41]
92	*Saccharum officinarum* L. Herb	Poaceae	Shankora ageda (Amh)	Fresh steam is put in the fire and eaten when gets hot to get relief from the common cold	Oral	C. cold	[48]
93	*Salix subserrata* Willd	Salicaceae		Crushed leaves of *Salix subserrata* Willd. and *Afrocarpus falcatus* (Thunb.) C. N. Page was also used in fresh form, mixed with water and milk, to treat rabies	Oral	Rabies	[73]
94	*Sesamum indicum*	Pedaliaceae	Selit (Amh)	two drops of sesame oil in each nostril each morning are suggested to prevent COVID-19	Nasal	COVID-19	CCRH, 2020
95	*Schinus molle*	Anacardiaceae	Selit (Amh) Kendo berberie (Amh)	Pounded Crushed Fruit	Oral	Cough (C. cold)	[36]
				Crushed fresh leaves of *Schinus mole* with water	Oral	H. zoster	[44]
96	*Solanecio gigas* (Vatke) C. Jeffrey	Asteraceae	Boz (Amh)	Leaves are collected from seven different areas, grounded with *Guizotia abyssinica* seeds, mixed with water and solution have taken orally	Orally	Hepatitis	[39]
97	*Sorghum bicolor* (L.) Moench.	Poaceae	Boz (Amh)	Boil it in water and wash the body with it	Dermal	H. zoster	[35]
98	*Spinacia oleracea*	Amaranthaceae	Keyh leqa (Tig)	—	—	SARS-CoV-2	[10]
99	*Stephania abyssinica* (Dillon & A. Rich.) Walp.	Menispermaceae	Kosta (Amh)	Crushed and given with milk and water	Orally	Rabies	[38]
100	*Syzygium aromaticum*	Myrtaceae	Chewchawit (Amh)	—	—	HSV-1 and 2	[9]
101	*Trichilia dregeana*	Meliaceae	Kirnfud (Amh)	Soaked, cooked, and put on tooth surface	dermal	Wound Warts	[36]
102	*Triumfetta heterocarpa* Sprague and Hutch.	Tiliaceae	Anunu (Oro)	The crushed fresh root is mixed with water and taken orally without food	Orally	Hepatitis	[49]
103	*Verbascum sinaiticum* Benth.	Scrophulariaceae	Yelam tut (Amh)	Roots are burned on fire and the smoke inhaled	Nasal	Hepatitis	[39]
104	*Vitis vinifera*	Vitaceae	Qetetina (Amh)	Fruits	Oral	HSV-1, PIV	[8]
105	*Vernonia amygdalina* Del.	Asteraceae	Weyin fire (Amh)	Leaves/roots	Oral	hepatitis, H. zoster, HSV, cough, HIV	[46]
106	*Warburgia ugandensis* Sprague	Canellaceae	Befit (Oro)	The smoke of 2-3 stick vascular part is inhaled to relieve cough	Nasal	Cough (C. cold)	[55]
107	*Withania somnifera*	Solanaceae	Giziewa or Kumo (Amh)	—	—	IBDV, HSV-1	[76]
				Fresh leaf and root will be crushed	Orally	Hepatitis	[36]
				Leaf and root crushed and drunk after boiling, powdered, juiced and drunk for 4 days, squeezed with leaves	Oral	Cough (C. cold)	[36]
108	*Ximenia americana* L.	Oleaceae	Enkuay (Amh)	Soaking bark in water and the water is taken orally	Orally	Rabies	[49]
109	*Zehneria scabra* (l.f.) Sond	Cucurbitaceae	Qorii Sinbiraa (Oro)	The pounded root of *Zehneria scabra* is concocted with the pounded root of *Ricinus communis* One feast of the pond is given to cattle and pack animals	Oral	Rabies	[41]
110	*Zingiber officinale* Roscoe.	Zingiberaceae	Zinjibile (Amh)	The stem is pounded well and boiled with water and drink	Orally, nasal	Influenza	[36, 37, 45]
				2–5 medium roots crushed and boiled with tea or water and then taken	Oral	Cough and c. cold	[43, 55]
111	*Ziziphus abyssinica* Hochst. ex A. Rich.	Rhamnaceae	Kurkura (Amh)	Fresh leaves and root are crushed and mixed with water and taken orally	Orally	Hepatitis	[49]

Notes: H. zoster = herpes zoster; C. cold = common cold; BCV = bovine coronavirus; HSV-1 = herpes simplex virus type 1; CVB1 = Coxsackie B virus type 1; IBDV = infectious bursal disease virus; RA = route of administration; DT = disease treated; Amh = Amharic; Oro = Oromo; Tig = Tigrinya; Afa = Afar; Had = Hadiyya; Sid = Sidaamu-afoo.

**Table 2:** Family and species groups of the reviewed medicinal plants.

No.	Family	Species per family		Medicinal plants per family	
		No. (%)	Rank	No. (%)	Rank
1.	Fabaceae	8 (7.2)	1	9 (5.6)	3
2.	Lamiaceae	6 (5.4)	2	9 (5.6)	3
3.	Alliaceae	2 (1.8)		8 (4.9)	4
4.	Phytolaccaceae	1 (0.9)		8 (4.9)	4
5.	Acanthaceae	3 (2.73)	4	7 (4.3)	5
6.	Myrtaceae	2 (1.8)		6 (3.7)	6
7.	Zingiberaceae	2 (1.8)		6 (3.7)	6
8.	Asteraceae	5 (4.5)	3	5 (3.09)	7
9.	Moraceae	3 (2.73)	4	5 (3.09)	7
10.	Anacardiaceae	3 (2.73)	4	4 (2.5)	8
11.	Apiaceae	3 (2.73)	4	3 (1.85)	
12.	Cucurbitaceae	3 (2.73)	4	3 (1.85)	
13.	Meliaceae	3 (2.73)	4	3 (1.85)	
14.	Oleaceae	3 (2.73)	4	3 (1.85)	
15.	Rutaceae	3 (2.73)	4	3 (1.85)	
16.	Vitaceae	3 (2.73)	4	3 (1.85)	
17.	Apocynaceae	2 (1.8)		3 (1.85)	
18.	Ranunculaceae	2 (1.8)		3 (1.85)	
19.	Amaranthaceae	2 (1.8)		2 (1.23)	
20.	Asclepiadaceae	2 (1.8)		2 (1.23)	
21.	Poaceae	2 (1.8)		2 (1.23)	
22.	Podocarpaceae	2 (1.8)		2 (1.23)	
23.	Polygonaceae	2 (1.8)		2 (1.23)	
24.	Rosaceae	2 (1.8)		2 (1.23)	
25.	Caricaceae	1 (0.9)		2 (1.23)	
26.	Musaceae	1 (0.9)		2 (1.23)	
27.	Papaveraceae	1 (0.9)		2 (1.23)	
28.	Santalaceae	1 (0.9)		2 (1.23)	
29.	Simaroubaceae	1 (0.9)		2 (1.23)	
30.	Theaceae	1 (0.9)		2 (1.23)	
31.	Solanaceae	6 (5.4)^∗2^		12 (7.41)	1
32.	Euphorbiaceae	5 (4.5)^∗3^		11 (6.8)	2
33.	Aloaceae	1 (0.9)		1 (0.6)	
34.	Amaryllidaceae	1 (0.9)		1 (0.6)	
35.	Araliaceae	1 (0.9)		1 (0.6)	
36.	Boraginaceae	1 (0.9)		1 (0.6)	
37.	Brassicaceae	1 (0.9)		1 (0.6)	
38.	Canellaceae	1 (0.9)		1 (0.6)	
39.	Combretaceae	1 (0.9)		1 (0.6)	
40.	Dipsacaceae	1 (0.9)		1 (0.6)	
41.	Lobeliaceae	1 (0.9)		1 (0.6)	
42.	Melianthaceae	1 (0.9)		1 (0.6)	
43.	Menispermaceae	1 (0.9)		1 (0.6)	
44.	Moringaceae	1 (0.9)		1 (0.6)	
45.	Myricaceae	1 (0.9)		1 (0.6)	
46.	Oliniaceae	1 (0.9)		1 (0.6)	
47.	Pedaliaceae	1 (0.9)		1 (0.6)	
48.	Piperaceae	1 (0.9)		1 (0.6)	
49.	Plantaginaceae	1 (0.9)		1 (0.6)	
50.	Polygalaceae	1 (0.9)		1 (0.6)	
51.	Rhamnaceae	1 (0.9)		1 (0.6)	
52.	Rubiaceae	1 (0.9)		1 (0.6)	
53.	Salicaceae	1 (0.9)		1 (0.6)	
54.	Sapindaceae	1 (0.9)		1 (0.6)	
55.	Scrophulariaceae	1 (0.9)		1 (0.6)	
56.	Tiliaceae	1 (0.9)		1 (0.6)	
57.	Trymalaceae	1 (0.9)		1 (0.6)	
	Total	111		162	

**Table 3:** Medicinal plants with antiviral components.

No.	Scientific name	Family	Local name	Active components	References
1	*Acacia abyssinica* Hochst.ex Benth.	Fabaceae	Bazra grar (Am)	Flavonoid, tannin, terpenoids, polyphenolic	[5]
2	*Acacia etbaica* Schweinf.	Fabaceae	Seraw (Am)	Flavonoid, tannin, terpenoids, polyphenolic	[5]
3	*Acacia nigra*	Fabaceae	Tikur grar (Am)	Flavonoid, tannin, terpenoids, and polyphenolic	[5]
4	*Acanthus polystachyus*	Acanthaceae	Kucheshile (Am)	Tannins, flavonoids, saponins, polyphenols, and anthraquinones	[78]
5	*Acokanthera schimperi*	Apocynaceae	Meriz (Am)	Oleanolic acid and ursolic acid	[79]
6	*Allium cepa*	Alliaceae	QeY shikurt (Am)	Quercetinand epigallocatechin gallate	[16]
7	*Allium sativum*	Alliaceae	Nechsenkret (Am)	Allicin, diallyl trisulfide ajoene, and apigenin	[14, 17]
8	*Aloe macrocarpa* Tod.	Aloaceae	Eret (Am)	Aloenin, aloesin, aloe-emodin, aloin chrysophanol, catechin, and isoaloresin	[18]
9	*Amaranthus hybridus* Linn.	Amaranthaceae	Tenbelel (Am)	Amaranthine, quercetin, and kaempferol glycosides	[80]
10	*Artemisia afra* Jack. ex Willd. and *Artemisia annua* L.	Asteraceae	Chikugn (Am)	Artemisinin	[6, 19]
11	*Azadirachta indica*	Meliaceae	Neem (Am)	Quercetin and ß sitosterol, polyphenolic flavonoids	[81]
12	*Bersama abyssinica*	Melianthaceae	Azamer (Am)	Anthraquinones	[82]
13	*Brassica carinata* A. Br. Herb	Brassicaceae	Gommon (Am)	Kaempferol	[10, 11]
14	*Camellia sinensis*	Theaceae	Shay kitel (Am)	Epigallocatechin gallate	[10]
15	*Capsicum annuum L.*	Solanaceae	Berbere (Am)	Apigenin	[11]
16	*Carissa edulis*	Apocynaceae	Agam (Am)	Kaempferol and quercetin	[83]
17	*Citrus aurantium L*	Rutaceae	Bahir Lome (Am)	Apigenin, ethyl cholate, nobiletin, tangeretin, chalcone, and hesperidin	[5, 10, 14, 15]
18	*Citrus limon (L.) Burm. f.*	Rutaceae	Lome (Am)	Apigenin, ethyl cholate, nobiletin, tangeretin, chalcone, and hesperidin	[5, 10, 14, 15]
19	*Clematis hirsute*	Ranunculaceae	Hareg (Tg)	Kaempferol and quercetin	[84]
20	*Clutia abyssinica*	Euphorbiaceae	Tewshealalito (Tg) Fiyle feji (Am)	Anthraquinones	[85]
21	*Coriandrum sativum*	Apiaceae	Dimblal (Am)	Linalool, geranyl acetate	[56]
22	*Crinum abyscinicum* Hochst. ex A. Rich	Amaryllidaceae	Yejib shinkurt (Am)	Lycorine	[57]
23	*Curcuma longa*	Zingiberaceae	Erd (Am)	Curcumin	[20]
24	*Dodonia angustifolia*	Sapindaceae	Kitkita (Am)	Anthraquinones	[86]
25	*Dregea schimperi*	Asclepiadaceae	Shanqoq (Tg)	Anthraquinones	[87]
26	*Ekebergia capensis*	Meliaceae	Sembo (Am)	Oleanolic acid	[88]
27	*Eucalyptus globulus*	Myrtaceae	Nech bahirzaf (Am)	Jensenone	[22]
28	*Euphorbia abyssinica* G.F.Gmel	Euphorbiaceae	Kulkual (Am)	Oleanolic acid	[89]
29	*Lepidium sativum*	Brassicaceae	feto (Am)	Kaempferol and quercetin	[22]
30	*Lycopersicon esculentum* (L.) Mill.	Solanaceae	Timaatima (Or)	Rhoifolin	[64]
31	*Moringa borziana Mattei Mawe*	Moringaceae	Tamergnaw ketel (Shiferaw) (Am)	Flavonoids, anthraquinone, and hydroxychloroquine	[21]
32	*Musa spp.*	Musaceae	Muz (Am)	Rhoifolin	[64]
33	*Nigella sativa*	Ranunculaceae	Tikur Azmud (Am)	Hederagenin, nigellidine, and *α*-hederin	[11, 90]
34	*Ocimum basilicum* L. Herb	Lamiaceae	Bessobla (Am)	Oleanolic acid and ursolic acid	[11]
35	*Ocimum lamiifolium* Hochst. Ex Benth.	Lamiaceae	Damakassie (Am)	Oleanolic acid and ursolic acid	[11]
36	*Ocimum urticifolium* Roth	Lamiaceae	Dama kesie (Am)	Oleanolic acid and ursolic acid	[11]
37	*Olea europaea* subsp. *cuspidate*	Oleaceae	Weyra (Am)	Oleanolic acid and ursolic acid	[11]
38	*Osyris quadripartite*	Santalaceae	Keret (Am)	Ursolic acid, oleanolic acid (triterpenes), kaempferol-3-O-rutinoside, quercetin-3-O-rutinoside or rutoside, and quercetin-3-O-*β*-D-glucopyranoside (flavonoids)	[91]
39	*Phaseolus vulgaris*	Fabaceae	Bakela (Am)	Kaempferol	[92]
40	*Phytolacca dodecandra*	Phytolaccaceae	Endod (Am)	Oleanolic acid	[93]
41	*Piper nigrum*	Piperaceae	Kundo berbere (Am)	Piperine	[12]
42	*Prunus dulcis*	Rosaceae	Lewuz (Am)	Almond oil	[94]
43	*Ricinus communis* L.	Euphorbiacea	Kabosimbiro (Or)	Kaempferol and quercetin	[95]
44	*Rosa abyssinica*	Rosaceae	Qega (Am)	Unknown	[74]
45	*Rosmarinus officinalis*	Lamiaceae	Tibs kitel (Am)	Rosmarinic acid	[11]
46	*Rumex abyssinicus*	Polygonaceae	Mekmoko (Am)	Anthraquinones	[96]
47	*Rumex crispus*	Polygonaceae	Enbacho (Am)	Anthraquinones	[96]
48	*Ruta chalepensis* L.	Rutaceae	Tena adam (Am)	Kaempferol and quercetin	[13]
49	*Schinus molle*	Anacardiaceae	Kendo berbera (Am)	Piperine	[12]
50	*Spinacia oleracea*	Amaranthaceae	Kosta (Am)	Kaempferol	[10]
51	*Syzygium aromaticum*	Myrtaceae	Kirnfud (Am)	Oleanolic acid	[9]
52	*Vernonia amygdalina*	Asteraceae	Grawa (Am)	Anthraquinones	[97]
53	*Vitis vinifera*	Vitaceae	Weyin fire (Am)	Resveratrol rhoifolin	[8]
54	*Withania somnifera*	Solanaceae	Giziewa or Kumo (Am)	—	[76]
55	*Ximenia americana*	Oleaceae	Enkuay (Am)	Anthraquinones	[98]
56	*Zingiber officinale* Roscoe.	Zingiberaceae	Zinjibile (Am)	Gingerol shogaol	[7]

## Data Availability

All related data have been presented within the manuscript. The dataset supporting the conclusions of this article is available from the authors on request.
